# Supplementary material for: k-mer manifold approximation and projection for visualizing DNA sequences
Source: Genome Res. 2025 May;35(5):1234–46. doi: 10.1101/gr.279458.124 (PMC12047656; doi:10.1101/gr.279458.124)
Supplement: Supplement 8 [file Supplemental_Data_1.zip › Supplemental_Data_1/FOXC2_TGAGTG30NTGA_AI_5/kmap_logo.pdf]

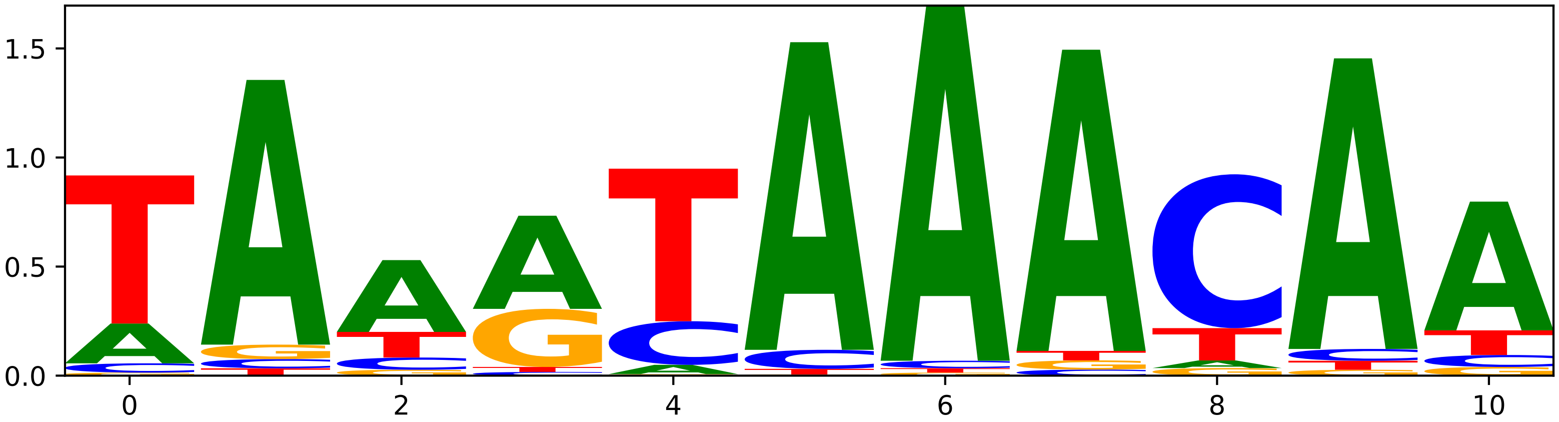
1.5
1.0
0.5
0.0
0
2
4
6
8
10
